# MicroRNA-155 facilitates skeletal muscle regeneration by balancing pro- and anti-inflammatory macrophages

**DOI:** 10.1038/cddis.2016.165

**Published:** 2016-06-09

**Authors:** M Nie, J Liu, Q Yang, H Y Seok, X Hu, Z-L Deng, D-Z Wang

**Affiliations:** 1 Department of Orthopaedic Surgery, The Second Affiliated Hospital, Chongqing Medical University, 76 Linjiang Road, Chongqing, P.R. China; 2 Department of Cardiology, Boston Children's Hospital, Harvard Medical School, 320 Longwood Avenue, Boston, MA, USA; 3 Animal Nutrition Institute, Sichuan Agricultural University, Chengdu, Sichuan, P.R. China

## Abstract

Skeletal muscle has remarkable regeneration capacity and regenerates in response to injury. Muscle regeneration largely relies on muscle stem cells called satellite cells. Satellite cells normally remain quiescent, but in response to injury or exercise they become activated and proliferate, migrate, differentiate, and fuse to form multinucleate myofibers. Interestingly, the inflammatory process following injury and the activation of the myogenic program are highly coordinated, with myeloid cells having a central role in modulating satellite cell activation and regeneration. Here, we show that genetic deletion of microRNA-155 (miR-155) in mice substantially delays muscle regeneration. Surprisingly, miR-155 does not appear to directly regulate the proliferation or differentiation of satellite cells. Instead, miR-155 is highly expressed in myeloid cells, is essential for appropriate activation of myeloid cells, and regulates the balance between pro-inflammatory M1 macrophages and anti-inflammatory M2 macrophages during skeletal muscle regeneration. Mechanistically, we found that miR-155 suppresses SOCS1, a negative regulator of the JAK-STAT signaling pathway, during the initial inflammatory response upon muscle injury. Our findings thus reveal a novel role of miR-155 in regulating initial immune responses during muscle regeneration and provide a novel miRNA target for improving muscle regeneration in degenerative muscle diseases.

Mammalian skeletal muscle is capable of repairing itself following exercise or injury. This remarkable regenerative capacity relies on satellite cells.^1, 2, 3, 4, 5^ Normally, satellite cells are kept underneath the basal lamina in a quiescent state. Upon muscle damage or disease, these quiescent stem cells immediately become activated, proliferate, migrate to the injured site, and differentiate to fuse with damaged myofibers or to form new myofibers.^1, 2, 3, 4^ The regeneration of adult skeletal muscle is a highly coordinated process involving a variety of cell types and signaling molecules that work systematically to repair the damaged myofibers.^2, 6, 7, 8^ However, how this process is regulated by muscle stem cell niche cues, such as inflammatory signals after muscle injury, still remains elusive.

Many stages of adult muscle regeneration are very similar to embryonic muscle development.^1, 9, 10, 11^ However, during adult muscle regeneration after acute injury, extrinsic factors are markedly different from those during embryonic development. The most notable and probably the most significant source of such extrinsic factors is the large number of inflammatory cells that infiltrate shortly after muscle damage.^8, 12, 13, 14, 15, 16^ It has been known that various inflammatory cells can profoundly affect the activation, migration, and differentiation of satellite cells, but the critical roles of inflammatory cells in maintaining skeletal muscle homeostasis have only recently begun to be appreciated.^8, 14, 16, 17^ Myeloid lineage cells, such as macrophages and the monocytes from which they are derived, are the major inflammatory cells recruited into injured skeletal muscle, and they are unique effector cells in innate immunity.^15, 16^ Following an early transient recruitment of neutrophils and mononuclear cells derived from circulating monocytes, these macrophages are primed by the inflammatory milieu, which includes local growth factors and cytokines, and begin to polarize into pro-inflammatory classically activated (M1-type) or anti-inflammatory alternatively activated (M2-type) macrophages, which differ in their markers, functions, and cytokine expression profiles.^8, 14, 15, 16, 18^ Normally, M1 macrophages first accumulate in the injured muscle tissues and produce high levels of inflammatory cytokines, which aid the clearance of apoptotic or necrotic cells and debris. The subsequent transition of myeloid infiltration into anti-inflammatory M2 macrophages is critical for the overall resolution of inflammation in the injured muscles.^8, 14, 15, 16, 18^ Therefore, loss of balance between these two different types of macrophages would severely compromise healing and regeneration of injured muscle.

miRNAs are small non-coding RNAs that are evolutionarily conserved from plants to mammals.^19^ Changes in miRNA expression have been associated with various muscle-wasting diseases, such as muscular dystrophies, and several miRNAs have been shown to exacerbate or prevent muscle disease progression in various mouse models of muscular dystrophies, and affect muscle regeneration.^20, 21, 22, 23, 24, 25, 26, 27, 28^ Furthermore, gain- and loss-of-function studies of miRNAs have clearly demonstrated their important roles in skeletal muscle regeneration and various muscle disorders.^20, 26, 27, 29, 30^ However, whether a miRNA can affect muscle regeneration by modulating myeloid cells in injured muscle is not well studied.

We have previously reported that microRNA-155 (miR-155) represses myogenic differentiation by targeting MEF2A, a key myogenic transcription factor, in C2C12 cells.^31^ Processed from the B-cell integration cluster gene (now designated the MIR-155 host gene or MIR-155HG), miR-155 is one of the best-characterized miRNAs, and numerous reports have indicated that miR-155 has a pivotal role in the immune system, particularly in hematopoietic cells upon virus or bacterial infection.^32, 33, 34, 35, 36, 37, 38^ However, despite rich knowledge about miR-155 in the immune system and several other systems,^39, 40, 41, 42^ the functions of miR-155 during myogenesis and muscle regeneration *in vivo* have not been determined. In this study, we found that miR-155 expression is substantially increased upon muscle injury and in *mdx* mice, the mouse model of Duchenne muscular dystrophy. Using genetic deletion of miR-155,^37, 38^ we showed that miR-155 facilitates skeletal muscle regeneration by regulating appropriate activation of macrophages. We found that miR-155-deficient mice exhibit delayed muscle regeneration, largely owing to aberrant macrophage activation and disrupted balance between the expression of pro- and anti-inflammatory cytokines. Together with a previous report on miR-21 function in macrophage transitions,^43^ our data support the notion that a macrophage-enriched miRNA can indirectly have profound effects on skeletal muscle regeneration, therefore providing a novel small RNA target for designing therapies for muscle injury and degenerative muscle diseases.

## Results

### Analysis of miR-155-knockout mice muscle phenotype

**Figure 1:**
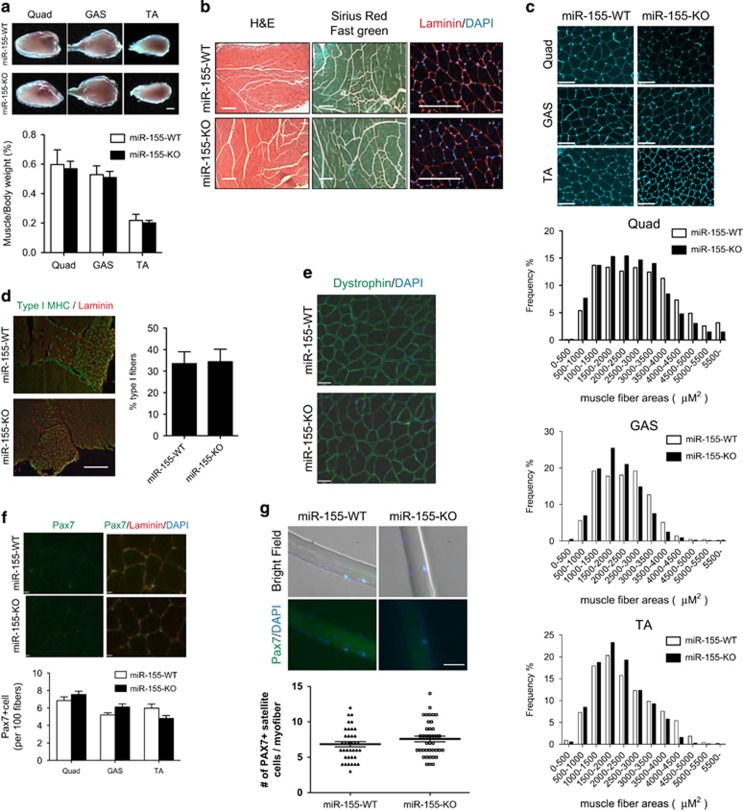
Characterization of skeletal muscle in miR-155-knockout mice. (**a**) Representative images and quantification of Quadriceps (Quad), Gastrocnemius (GAS), and Tibias anterior (TA) muscles of wild-type and miR-155-KO mice. Muscle weight was normalized to body weight. Bar, 2 *μ*m. (**b**) H&E staining, Sirius red/fast green collagen staining, and Laminin (red) and DAPI (Blue) immunostaining of cryosections of 8-week-old wild type and miR-155-KO TA muscle. Bar, 200 *μ*m. (**c**) Wheat-germ agglutinin (WGA) staining of muscle membranes in Quad, GAS, and TA muscles of 8-week-old miR-155-knockout and wild-type control mice. Bar, 32 *μ*m. (**d**) Immunostaining of type I fibers (green) in soleus muscle of miR-155-knockout mice and their wild-type littermate controls. Laminin (red) is stained to delineate the muscle fibers. Bar, 500 *μ*m. (**e**) Dystrophin immunostaining analysis of the Gastrocnemius muscle from miR-155-knockout mice and wild-type controls revealed normal sarcolemmal localization of dystrophin. Bar, 47 *μ*m. (**f**) Immunostaining of Pax7 (green) and Laminin (red) to identify satellite cells in skeletal muscle cross-sections of miR-155-knockout and wild-type control mice. Satellite cell numbers in Quad, GAS, and TA muscles (*n*=6 for each group) are quantified. Bar, 16 *μ*m. (**g**) Immunostaining for Pax7 on isolated single muscle fibers from EDL muscles of miR-155-knockout and wild-type control mice. Myofibers were isolated from three animals for each genotype, and 40 myofibers were measured for associated satellite cells. Data are presented as Mean±S.E.M. Bar, 30 *μ*m. Unless specifically marked, all results were obtained from eight mice for each genotype and data are presented as Mean±S.E.M.

We have previously reported that overexpression of miR-155 represses C2C12 myogenic differentiation through inhibition of MEF2A.^31^ We thus chose to further assess the *in vivo* function of miR-155. The gross morphology and weight of skeletal muscle in miR-155-KO mice are indistinguishable from that of their wild-type littermates (Figure 1a). Histological analysis reveals no obvious defects in the skeletal muscle of miR-155-KO mice. Similarly, no apparent fibrosis or degenerating muscle fibers are present in miR-155-KO mice (Figure 1b).

Next, we carefully examined fiber size and fiber type composition in miR-155 mice. We found no significant differences between miR-155-KO and control wild-type mice. Specifically, the myofiber cross-sectional area distributions of tibias anterior (TA), quadriceps (Quad) and gastrocnemius (GAS) muscles are very similar between miR-155-KO mice and wild-type controls (Figure 1c). Similarly, comparable percentages of type I myofibers were identified in the soleus muscle of miR-155-KO and wild-type mice (Figure 1d). Dystrophin immunostaining showed normal sarcolemmal membranes in miR-155-KO muscle (Figure 1e) suggesting normal sarcolemma integrity. Finally, a similar number of Pax7-positive satellite cells were observed in cross-sections of TA muscles of miR-155-KO and WT mice (Figure 1f). Alternatively, similar numbers of Pax7^+^ satellite cells associated with isolated single myofibers from EDL of miR-155-KO and wild type mice (Figure 1g). Together, these data demonstrate that loss of miR-155 does not affect normal skeletal muscle development nor affect satellite cell numbers.

### Delayed muscle regeneration in miR-155-KO mice

**Figure 2:**
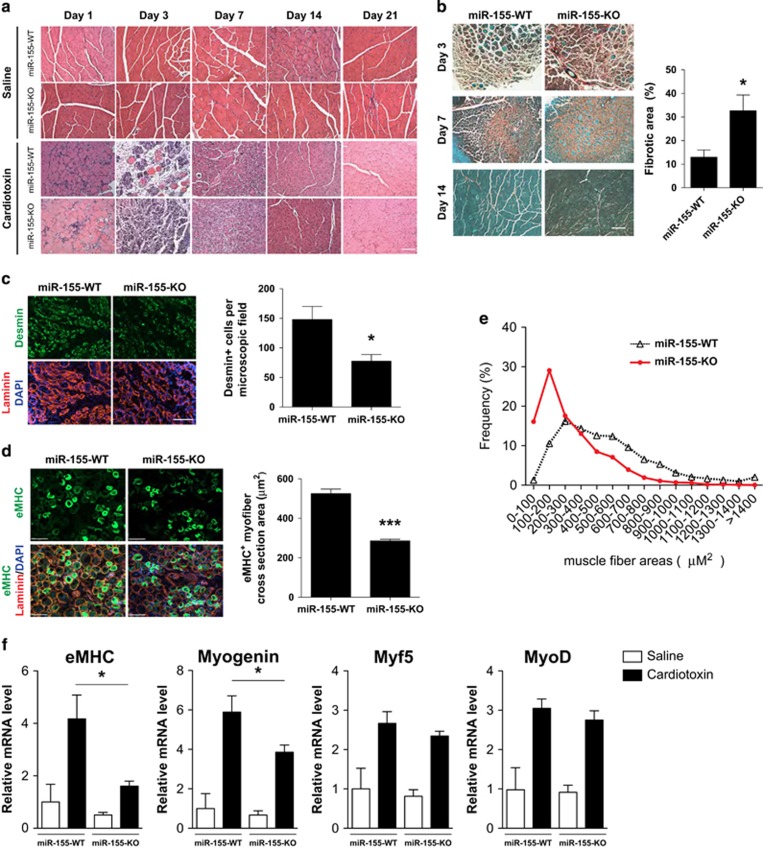
Delayed muscle regeneration in miR-155-knockout mice after cardiotoxin (CTX) injection. (**a**) H&E staining of transverse sections of WT and miR-155-KO TA muscles at day 1, 3, 7, 14, and 21 after CTX injury. Bar, 100 *μ*m. (**b**) Sirius Red/Fast Green staining of collagen in transverse sections of WT and miR-155-KO TA muscles at day 3, 7, and 14 days after CTX injury. Fibrotic areas were quantified in ImageJ based on Sirius Red staining. Bar, 200 *μ*m. Eight animals of each genotype were measured. *P<0.05. (**c**) Immunostaining for desmin and laminin on WT and miR-155-KO TA muscles at day 3 after CTX injury. Bar, 100 *μ*m. Number of desmin-positive fibers was quantified in ImageJ based on laminin staining. Four animals of each genotype were measured. *P<0.05. (**d**) Immunostaining for embryonic myosin heavy chain (eMHC) and laminin in WT and miR-155-KO TA muscles at day 7 after CTX injury. Bar, 75 *μ*m. Cross-sectional areas of regenerating eMHC-positive fibers were measured using ImageJ based on laminin staining. Only eMHC-positive fibers were measured. Four animals of each genotype and at least 500 fibers for each animal were measured. ***P<0.0001. (**e**) Distribution of the cross-sectional areas of eMHC-positive fibers of WT (red circle) and miR-155-KO (black open triangles) TA muscles at day 7 after CTX injury. Data are quantified from four animals for each genotype. (**f**) Real-time PCR showed less expression of late myogenic markers such as eMHC and myogenin but not early myogenic markers such as Myf5 and myoD in TA muscle of miR-155-KO mice 3 days after CTX injury. Six animals were measured for each genotype and data are presented as Mean±S.E.M.

Next, we examined whether miR-155 regulates the regeneration of skeletal muscle. We used the well-characterized cardiotoxin-induced injury model.^44^ Histological analysis at various time points after cardiotoxin injection revealed delayed regeneration of TA muscle in miR-155-knockout mice (Figure 2a). We found that the newly generated muscle fibers of miR-155-KO mice are smaller when compared with wild-type fibers at both 14 and 21 days post cardiotoxin injection (Figure 2a). Furthermore, Fast green and Sirius red staining revealed a significantly higher amount of fibrosis/necrosis in miR-155-KO mice as compared with wild-type controls 7 days post cardiotoxin injection (Figure 2b).

During muscle regeneration, newly formed muscle cells can be identified by their expression of muscle-specific proteins such as desmin, an intermediate filament protein that is abundantly expressed in skeletal muscle cells.^45^ Newly formed myofibers also differ from adult myofibers in their expression of the embryonic isoform of myosin heavy chain (eMHC).^46^ We observed fewer desmin-positive cells in miR-155-KO muscle three days post injury, suggesting delayed or diminished myogenic cell expansion (Figure 2c). Likewise, the cross-sectional areas of newly formed eMHC-positive myofibers are significantly smaller in miR-155-KO mice (Figure 2d). There is also an apparent skewing of the fiber size distribution toward smaller myofibers in miR-155-KO mice 7 days post cardiotoxin injection (Figure 2e). Accordingly, we also found decreased eMHC and myogenin transcript expression in miR-155-knockout mice 7 days post injury (Figure 2f). Interestingly, the expression of MyoD and Myf5, two early myogenic transcription factors that mark proliferating myoblasts, was not altered in miR-155-KO muscle (Figure 2f). Together, our results suggest that loss of miR-155 in mice significantly impairs the regeneration of adult skeletal muscle.

### Characterization of miR-155-KO primary myoblasts

**Figure 3:**
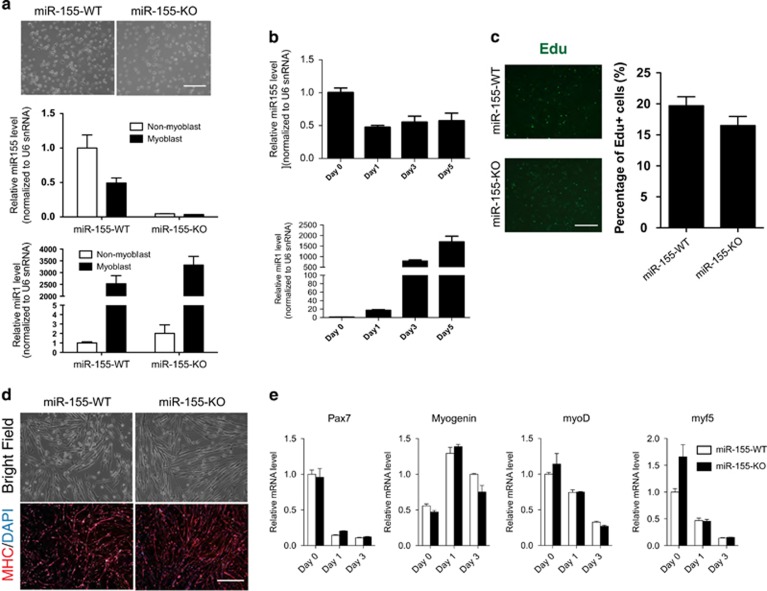
Loss of miR-155 does not affect satellite cell numbers, proliferation, or myogenic differentiation. (**a**) Phase contrast images show similar morphology of primary myoblasts isolated from miR-155-KO and control mice (upper). The expression of miR-155 and miR-1 in primary myoblasts and non-myoblasts (adherent cells of 1st preplate of myoblast isolation) isolated from neonatal miR-155-KO and control mice was measured by Taqman real-time PCR assays (lower). Bar, 200 *μ*m. (**b**) The expression of miR-155 and miR-1 was measured during primary myoblast differentiation. (*n*=3 per group). (**c**) Proliferation of primary myoblasts from miR-155-KO and control mice was measured using an EDU incorporation assay. Data are from three independent experiments and are presented as Mean±S.E.M. (**d**) Primary myoblasts from miR-155-KO and control mice were cultured in differentiation medium for 3 days and myogenic differentiation was determined by immunostaining against MHC. Bar, 200 *μ*m. (**e**) Pax7, myoD, Myf5, and myogenin expression was measured by real-time PCR. Data are from three independent experiments and are presented as Mean±S.E.M.

The apparent defects in muscle regeneration upon injury in miR-155-KO mice prompted us to examine whether the activation and differentiation of satellite cells are affected. Primary myoblasts were isolated from neonatal miR-155-KO or wild-type control mice, along with non-myogenic cells, largely consisting of fibroblasts, endothelial cells, and mesenchymal stromal cells.^47^ Surprisingly, we observed that miR-155 is not enriched in primary myoblasts; rather, it is expressed more in non-myogenic cells. This is in sharp contrast with miR-1, a miRNA enriched in myoblasts (Figure 3a). We also found that miR-155 levels decreased during differentiation to about half of its original level (Figure 3b). In addition, we also observed a similar expression profile of miR-155 in C2C12 (Supplementary Figure S1D). Furthermore, we were unable to detect activity of the beta-galactosidase reporter gene, which was an initial knock-in at the miR-155 locus, in adult skeletal muscle (data not shown), supporting low endogenous miR-155 expression in adult skeletal muscle. Loss of miR-155 did not appear to affect cell proliferation of isolated primary myoblasts, as measured by EdU incorporation assays (Figure 3c). In addition, primary myoblasts isolated from miR-155-KO mice differentiate into multinuclear myofibers as robust as those of wild-type controls, suggesting that miR-155 is dispensable for myoblast differentiation (Figure 3d). These observations are further supported by results demonstrating no alteration of the expression of myogenic factors in miR-155-KO myoblasts (Figure 3e). Together, these results indicate that miR-155 is expressed at extremely low levels in muscle cells *in vivo,* such that suppression of its targets, such as MEF2A, could only be detected when miR-155 is overexpressed in C2C12 cells.^31^

### Inflammatory cytokine expression was altered in injured skeletal muscle of miR-155-KO mice

**Figure 4:**
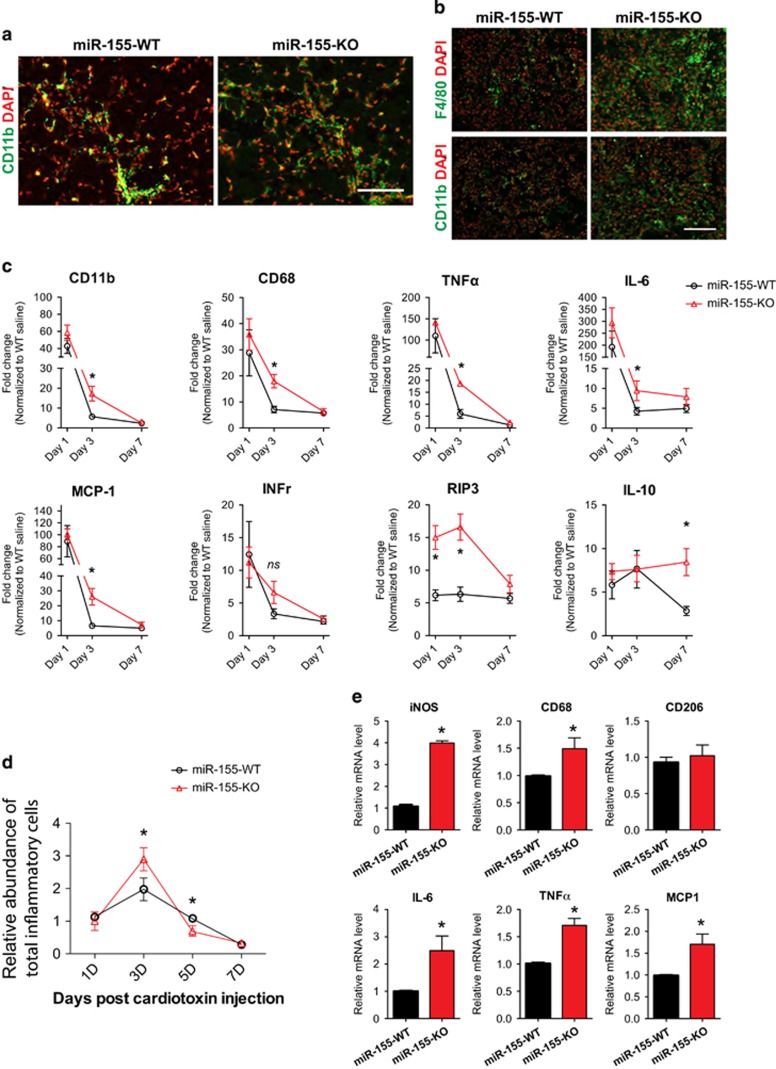
Aberrant immune response of miR-155-KO skeletal muscle after injury. (**a**) Immunostaining for CD11b (green) and DAPI (red) on transverse sections of miR-155-WT and miR-155-KO TA muscles 1 day after CTX injury. Bar, 50 *μ*m. (**b**) Immunostaining for F4/80 or CD11b (green) and DAPI (red) on transverse sections of miR-155-WT and miR-155-KO TA muscles 3 days after CTX injury. Bar, 100 *μ*m. (**c**) Real-time PCR for expression of CD11b, CD68, TNF*α*, IL-6, MCP-1, INF*γ*, RIP3, and IL-10 in miR-155-WT and miR-155-KO TA muscles 1, 3, and 7 days after CTX injury. Data are quantified from six animals for each genotype and are presented as Mean±S.E.M. *P<0.05. (**d**) Relative numbers of inflammatory cells (F4/80- and CD11b-positive cells isolated using MACS) from miR-155-WT (black circle) and miR-155-KO (red triangle) TA muscles 1, 3, 5, and 7 days after CTX injury. Four to six animals of each genotype were quantified and data are presented as Mean±S.E.M. (**e**) Real-time PCR for expression of iNOS, CD68, CD206, IL-6, TNF*α*, and MCP-1 in MACS-isolated macrophages from miR-155-WT and miR-155-KO TA muscles 3 days after CTX injury. Four to six animals of each genotype were quantified and data are presented as Mean±S.E.M. *P<0.05

The above seemingly contradictory results, in which miR-155-KO muscle displayed defects in muscle regeneration, yet isolated miR-155-null primary myoblasts exhibited normal cell proliferation and differentiation, provoked us to examine whether miR-155 may function in non-muscle cells. Specifically, we hypothesized that the immune response that occurs naturally after muscle damage may be affected in miR-155-KO mice. We first assessed whether there is an altered immune response when miR-155 expression is abolished. Similar amounts of CD11b-positive cells are seen infiltrating damaged skeletal muscles 1 day after injection (Figure 4a). However, more F4/80^+^ macrophages are observed 3 days post cardiotoxin injection in miR-155-knockout mice (Figure 4b). Pro-inflammatory cytokines (TNF-a, IL-6, INFr, MCP-1) were present in higher levels over time in miR-155-KO muscle after injury, suggesting a delay in the clearance of M1-type inflammatory macrophages. Conversely, the anti-inflammatory cytokine IL-10 and the necrotic marker gene RIP3 was present at a lower level 7 days post injury compared with wild type, suggesting a delay in the activation of M2-type macrophages (Figure 4c). We also utilized MACS to isolate and quantify macrophages with different markers (F4/80 and/or CD11b) after cardiotoxin injection. Consistent with our immunostaining results, there are substantially more inflammatory immune cells (macrophages/monocytes/leukocytes) in miR-155-KO mice 3 days after injury when compared with wild-type littermate controls (Figure 4d). In addition, RT-qPCR of isolated cells confirmed the changes in expression of cytokines and macrophage markers, including iNOS, CD68, CD206, IL-6, TNF*α*, and MCP-1 (Figure 4e). These results indicate that the conversion from pro- to anti-inflammatory macrophages is delayed in miR-155-KO mice after muscle injury, which likely explains the observed delay in muscle regeneration in miR-155-KO mice.

### Altered balance between pro- and anti-inflammatory macrophages in miR-155-KO skeletal muscle after injury

**Figure 5:**
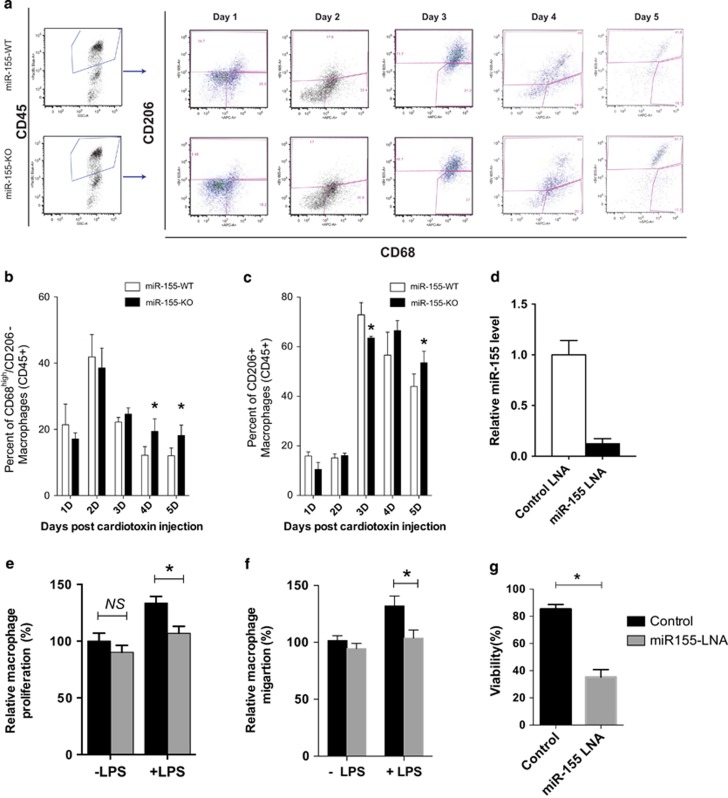
miR-155-knockout prolongs the pro-inflammatory stage and delays the anti-inflammatory phase of macrophages in skeletal muscle after injury. (**a**) Representative FACS analysis of M1 (CD45+CD68^high^CD206^−^) and M2 (CD45^+^CD68^−^CD206^+^) type macrophages in the TA muscles of miR-155-WT and miR-155-KO mice 1, 2, 3, 4, and 5 days after CTX injury. For each day, the same gating criteria were applied for pair-wise comparisons. (**b**) Quantification of the percentage of M1-type macrophages in miR-155-WT and miR-155-KO TA muscles 1–5 days after CTX injury. *P<0.05 (**c**) Quantification of the percentage of M2-type macrophages in miR-155-WT and miR-155-KO TA muscles 1–5 days after CTX injury. *P<0.05. For all FACS analyses, four to six animals were measured for each genotype at each time point. (**d**) Mouse macrophage RAW 264.7 cells were transfected with control and miR-155 LNA (locked nucleic acid) inhibitor at a final concentration of 30 nM. Levels of miR-155 in transfected cells were determined by Taqman real-time PCR assays. (**e**) Cell proliferation was measured 36 h after transfection using Click-iT EdU imaging kit (Invitrogen). (**f**) Cell migration was assessed using BD 24-well Transwell assays 48 h after transfection. (**g**) Cell viability was determined using Invitrogen Countess automatic cell counter 72 h after LNA transfection. Data are presented as Mean±S.E.M. *P<0.05

To further understand which macrophage types are affected in miR-155-knockout mice after muscle injury, we monitored and quantified M1 and M2 macrophage populations after cardiotoxin injection every day for 5 days, using FACS (Figure 5a). Using CD68 and CD206 as reliable markers for M1/M2 macrophages,^16, 48, 49, 50, 51^ we measured the relative abundance of M1 and M2 macrophages residing in the TA muscle after cardiotoxin-mediated injury in miR-155-KO and control mice. Total leukocytes were first sorted by positive staining for CD45, a widely used leukocyte marker.^52, 53^ Percentage of M1 (CD68^high^/CD206−) or M2 (CD206+) macrophages were quantified by FACS analysis (Figure 5a, b and c). Our results showed a prolonged M1 phenotype retention phase (Figure 5b) and corresponding delayed M2 activation in miR-155-KO muscle (Figure 5c). More specifically, the M1 macrophage portion of total leukocytes in miR-155-KO muscle was significantly higher than that in wild-type controls 4–5 days after cardiotoxin injection (Figure 5b). Conversely, the M2 macrophage proportion was significantly less in miR-155-knockout mice than that in wild-type mice 3 days post muscle injury; however, it exceeded that of wild-type mice 5 days post injury, when the total leukocyte number had started to rapidly decrease (Figure 5c). These data suggest that the conversion from pro-inflammatory to anti-inflammatory macrophages is delayed in miR-155-KO muscle (Figure 5b and c). To further confirm the role of miR-155 in macrophages, we performed *in vitro* macrophage proliferation, migration, and viability assays using the widely used Raw 264.7 mouse macrophage cell line. Transient transfection of LNA against miR-155 effectively knocked down the endogenous miR-155 level (Figure 5d). Inhibition of miR-155 resulted in reduced macrophage proliferation (Figure 5e) and migration (Figure 5f) after LPS activation. Loss of miR-155 also reduced the viability of macrophages (Figure 5g). Taken together, our data indicate that miR-155 regulates the balance of pro- and anti-inflammatory macrophages during muscle regeneration by directly modulating macrophage proliferation, migration, and survival.

### miR-155 regulates macrophage activity by targeting SOCS1 and the JAK-STAT signaling pathway

**Figure 6:**
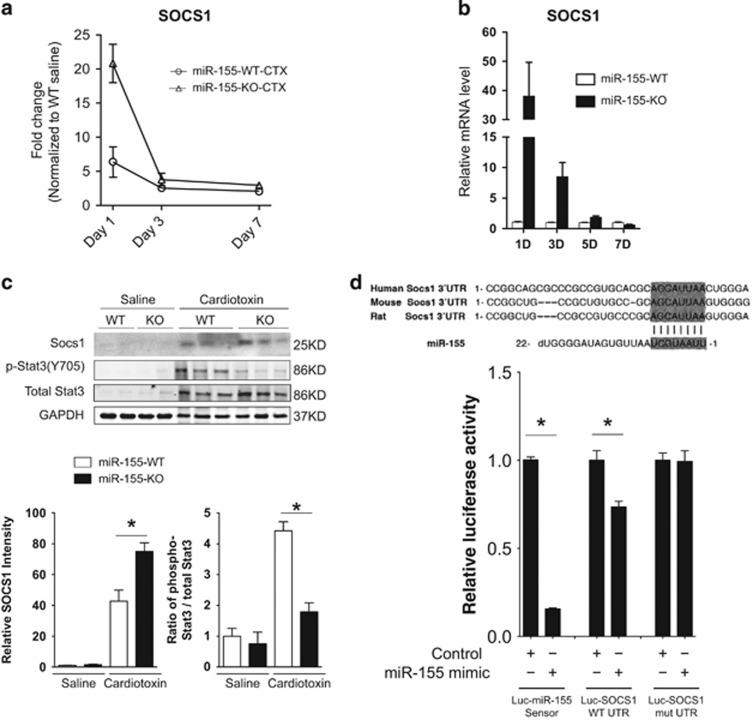
miR-155 targets SOCS1 in macrophages after skeletal muscle CTX injury to inhibit activation of STAT3 signaling. (**a**) Real-time PCR analysis of SOCS1 expression levels in miR-155-WT and miR-155-KO TA muscle 1, 3, and 7 days after CTX injury. Four to six animals of each genotype were quantified at each time point and data are presented as Mean±S.E.M. *P<0.05. (**b**) Relative expression of SOCS1 in MACS-isolated macrophages from miR-155-KO TA muscle normalized to miR-155-WT controls at 1, 3, 5, and 7 days after CTX injury. Four animals for each genotype and time point were analyzed and data are presented as Mean±S.E.M. *P<0.05. (**c**) Western blot analysis of SOCS1 and phospho-STAT3 levels in TA muscles 1 day after CTX injections, showing increased SOCS1 and decreased phospho-STAT3(Y705) signaling in miR-155-KO mice compared with wild type. The intensity of western blot bands was quantified by Imagestudio software (Odyssey) and was normalized to GAPDH for total protein loading. Two independent experiments were performed with similar results. Data are presented as Mean±S.E.M. *P<0.05. (**d**) Diagram showing the first conserved binding site of mmu-miR-155 on the 3′-UTR of mmu-SOCS1 highlighted. miR-155 directly represses the SOCS1 3′UTR in Luciferase assays, and repression is abolished when all three miR-155-binding sites are mutated. Triplicate biological samples were used in the luciferase assays. Data are presented as Mean±S.E.M. *P<0.05

Our previous studies have shown that miR-155 targets MEF2A and Jarid2 in skeletal muscle C2C12 cells and the heart, respectively.^31, 42^ Surprisingly, we found that the expression of these transcripts was not significantly altered in the skeletal muscle of miR-155-KO mice (Supplementary Figure S1A), suggesting that other targets likely mediate the effects of miR-155 in muscle regeneration. Indeed, miR-155 has been reported to directly target important signal transduction regulatory molecules such as SHIP1, TAB2, and suppressors of cytokine signaling 1 (SOCS1), all critical for M1 and M2-type macrophage activation and homeostasis.^33, 41, 54, 55, 56, 57^ We observed an increase in both SOCS1 and TAB2 mRNA levels in TA muscle after muscle injury (Figure 6a and Supplementary Figure S2). Interestingly, although there is an approximately fourfold increase in the SOCS1 mRNA level in the TA muscle of miR-155-KO mice 1 day after cardiotoxin injury (Figure 6a), the increase in SOCS1 is much more pronounced in MACS-isolated macrophages from miR-155-KO mice (Figure 6b). In addition, SOCS1 mRNA levels in miR-155-KO macrophages remain higher than those in wild-type controls throughout the initial muscle regeneration period (Figure 6b).

SOCS1 protein levels were also higher in TA muscle in miR-155-KO mice than in wild-type controls (Figure 6c, upper panel). Consistent with its previously reported role in inhibiting the JAK-STAT signaling pathway,^58^ we found that increased SOCS1 in miR-155-KO muscle effectively reduced the level of phospho-STAT3 immediately (1 day) after cardiotoxin injury (Figure 6c). Although at 3 days post injury the levels of phospho-STAT3 (Y705) phosphorylation appeared to be similar between miR-155-WT and miR-155-KO (Supplementary Figure S2), the level of phospho-STAT3 (S727), a downstream event of phospho-STAT3 (Y705),^59^ was increased in miR-155-KO muscle as compared with wild-type controls (Supplementary Figure S2), suggesting that the activation of STAT signaling and its subsequent transcriptional activity are delayed in miR-155-KO muscle after injury. Finally, using luciferase reporter assays, we found that miR-155 directly represses a luciferase reporter linked to the mouse SOCS1 3′-UTR, whereas mutation of all three potential miR-155-binding sites in the mouse SOCS1 3′-UTR completely abolished this repression (Figure 6d). Together, our results demonstrate that loss of miR-155 leads to SOCS1 upregulation in macrophages upon muscle injury, which in turn delays the activation of the JAK-STAT signaling pathway and the timely activation and transition of macrophages.

### Local administration of miR-155 restores normal muscle regeneration in miR-155-KO mice after injury

**Figure 7:**
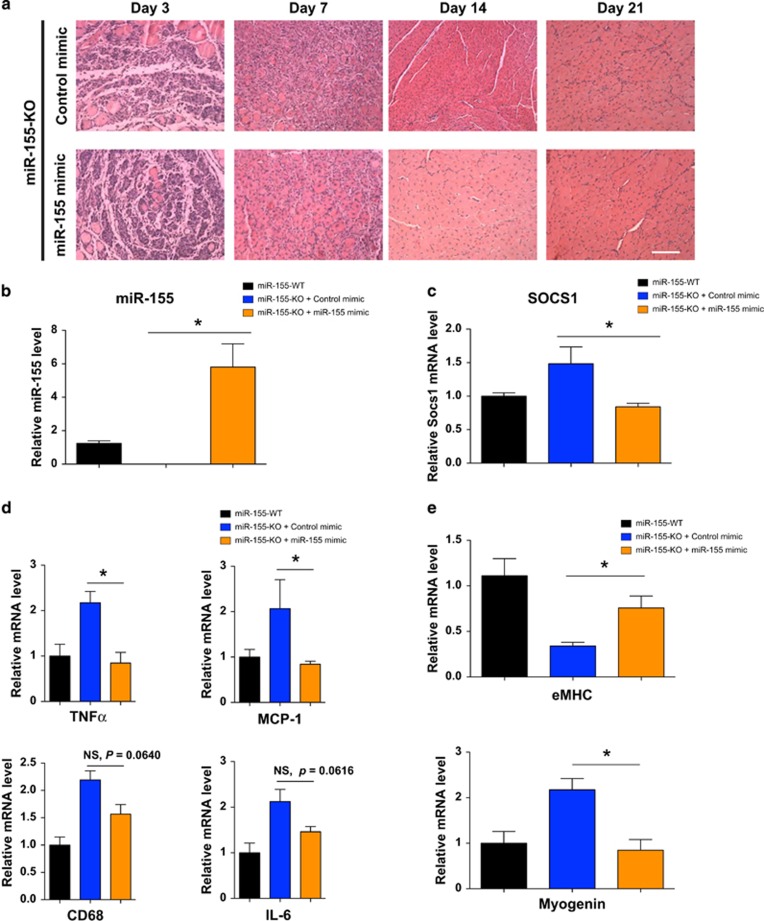
Overexpression of miR-155 mimic in TA muscle after CTX injury rescues muscle regeneration defects in miR-155-KO mice. (**a**) H&E staining of transverse sections of TA muscle from miR-155-KO with or without miR-155 mimic injection at 3, 7, 14, and 21 days after CTX injury. Bar, 100 *μ*m. (**b**) miR-155 expression was measured by Taqman real-time PCR assays 3 days after CTX injury and miRNA mimic injection. Four to six animals in each condition were analyzed and data are presented as Mean±S.E.M. *P<0.05. (**c**) SOCS1 mRNA levels were measured by real-time PCR in TA muscle of control mice and miR-155-KO mice injected with miRNA control mimic or miR-155 mimic 3 days after CTX injury. Injection of miR-155 mimic effectively inhibits SOCS1 levels in miR-155-KO mice. Four to six animals in each condition were analyzed and data are presented as Mean±S.E.M. *P<0.05. (**d**) Real-time PCR quantification of inflammatory markers (TNF*α*, MCP-1, CD68, IL-6) that are affected in miR-155-KO mice after CTX injury, showing restoration of these delayed macrophage activation markers after miR-155 mimic injection. (**e**) Real-time PCR shows that delayed eMHC and myogenin expression in miR-155-KO TA muscles 3 days after CTX injury is normalized when miR-155 mimic was injected compared with control miRNA mimic

We next tested whether restoring the acutely high expression of miR-155 locally could rescue macrophage homeostasis and prevent muscle regeneration defects. miR-155 or control mimics were injected in the TA muscle of one leg of miR-155-KO mice as previously described.^29, 60, 61^ By optimizing the concentration and time of injection after cardiotoxin injury, we were able to achieve close to physiological levels of miR-155 after injury (Figure 7b). Overexpression of miR-155 rescued the muscle regeneration defects of miR-155-KO mice after cardiotoxin injection, as evidenced by similar inflammatory cell infiltration and fiber sizes when compared with wild-type mice at day 7 and 14 (Figure 7a). Furthermore, the miR-155 target SOCS1 is also significantly repressed to comparable levels as wild-type mice after cardiotoxin injury (Figure 7c). We also examined the expression levels of the inflammatory cytokines and regulatory genes that were altered in miR-155-KO mice after miR-155 mimic injection. As expected, two of the cytokines, TNF*α* and MCP-1, showed significant downregulation in miR-155 mimic injected samples, and were nearly at the same level as in the wild-type mice (Figure 7d). Two other deregulated pro-inflammatory markers, CD68 and IL-6, also showed noticeable trends of downregulation, although they did not reach statistical significance (Figure 7d). Finally, we found that levels of eMHC and myogenin were restored after miR-155 mimic injection, further confirming that reintroducing miR-155 after muscle injury can fully rescue muscle regeneration defects (Figure 7e). Together, these results demonstrate that miR-155 is required by macrophages to mediate normal initial muscle regeneration *in vivo*.

## Discussion

**Figure 8:**
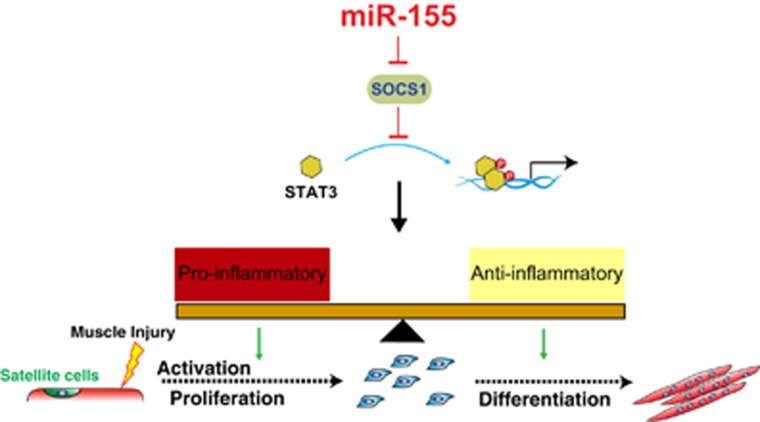
Schematic representation of miR-155 regulating the macrophage phenotype transition in skeletal muscle regeneration following injury. Following acute muscle injury, the early stage of muscle repair is characterized by the initial infiltration of neutrophils and monocytes, followed by the activation of pro-inflammatory macrophages, which coincides with the activation of quiescent satellite cells and their subsequent proliferation. As muscle repair proceeds, activation of miR-155 expression releases the SOCS1-mediated inhibition of Jak-STAT signaling, accompanied by a shift to anti-inflammatory macrophages. In the absence of miR-155, SOCS1 levels are elevated, thus dampening the Jak-STAT signaling that is critical for the balance of pro- and anti-inflammatory immune responses

The results of our study reveal an important role of miR-155 in regulating efficient skeletal muscle regeneration in response to injury and disease. Whereas mice lacking miR-155 do not exhibit overt abnormalities, lack of miR-155 in mice causes a significant delay in early skeletal muscle regeneration upon cardiotoxin injury, largely due to misregulation of the inflammatory response. Mechanistically, we revealed that miR-155 promotes muscle regeneration by regulating the balance between M1 and M2 macrophage activation and phenotype transition after muscle injury, at least in part by repressing its target SOCS1 and modulating the activity of the JAK-STAT signaling pathway (Figure 8). To our knowledge, this is the first time that a non-muscle-enriched miRNA has been implicated in adult skeletal muscle regeneration. At last, our results also extend the notion that miRNAs normally function as ‘fine-tuners' of biological phenotypes under tissue homeostasis, whereas their tissue-specific functions become prominent and magnified under stress conditions or tissue injuries.

Muscle regeneration requires intricate interactions between satellite cells and various other cell types that arise after muscle injury.^8, 13, 14, 62^ Timely and appropriate execution of each of these processes is critical for full recovery of the injured muscle. Much of the work on miRNAs in muscle regeneration have focused on studying satellite cell biology and on identifying novel miRNAs that affect their activation, proliferation, and myogenic differentiation.^30, 63, 64, 65^ Nevertheless, it is now clear that complex features of the microenvironment in which regeneration occurs can also determine the success or failure of muscle regeneration.^17^ Our results showing that the temporal activation and balance of M1 and M2-type macrophages are disrupted in miR-155-knockout mice, which accounts for the significant delay in muscle regeneration. In particular, we observed a prolonged M1 immune response and slightly delayed M2 macrophage appearance in injured muscle tissues in miR-155-knockout mice, suggesting a defect in coordinating the biphasic transition of macrophages. Along with several recent reports on macrophages in heart and skeletal regeneration,^51, 62, 66, 67, 68, 69^ our data demonstrate that accurate and faithful activation of macrophages after injury is of critical importance for muscle regeneration. Therefore, we propose that the defects of muscle regeneration we observed in miR-155-knockout mice predominately result from deregulated macrophage activation upon muscle injury (Figure 8).

miR-155 was among the first miRNAs to be linked to inflammation, owing to its potent upregulation in multiple immune cell lineages upon activation.^35, 38, 70, 71^ A wide variety of miR-155 targets relevant to mammalian immunity have been reported in various biological contexts.^32, 33, 34, 35, 37, 38, 40, 55, 72, 73^ Whether these mechanisms are also conserved and exist in myeloid cell lineages that infiltrate into skeletal muscle tissues after acute injury *in vivo* has not been determined. In the present study, we demonstrated that miR-155 directly targets SOCS1 and downstream JAK-STAT signaling in polarized macrophages residing in injured skeletal muscle tissue. Furthermore, we also demonstrated that miR-155 is critical for the delicate balance between pro- and anti-inflammatory macrophages after acute muscle injury. Although previous *in vitro* studies have demonstrated that miR-155 is important for the activation of pro-inflammatory (M1-type) macrophages,^73, 74^ our data provide direct evidence that miR-155 facilitates the activation of JAK-STAT signaling during the transition of M1 to M2 macrophages *in vivo*. Consistent with our observations in skeletal muscle, several other studies have revealed the functions of miR-155 in the immune responses of macrophages in a variety of other tissues.^33, 40, 73^ Altogether, our results provide new insights into how miR-155, a miRNA enriched in activated macrophages, affects myogenic regeneration after muscle injury and modulates timely muscle repair by controlling the delicate balance of pro- and anti-inflammatory macrophage activation.

Duchenne muscular dystrophy is a progressive, lethal, muscle-wasting disease caused by a null mutation in the dystrophin gene.^75^ In the absence of dystrophin, sarcolemmas become fragile and weak, causing repeated myofiber injury. Most studies on miRNA function in muscle diseases have been focused on identifying deregulated miRNAs using genome-wide analyses. Several of them have been confirmed to be able to negative or positively affect muscle disorders or the progression of myopathy *in vivo*.^20, 23, 25, 27, 30, 76, 77^ Intriguingly, miR-155 is among the miRNAs that are deregulated in human muscle disorders.^22^ Accordingly, results of our present study also demonstrate the upregulation of miR-155 upon muscle injury and in *mdx* muscles, implying its involvement in muscle diseases. It remains to be determined whether aberrant miR-155 expression in dystrophic muscle directly causes chronic inflammation and disrupts macrophage homeostasis. It will also be essential to determine how misregulation of the immune response owing to lack of miR-155 in dystrophic muscle contributes to the pathogenesis of the disease. The active role of miR-155 in regulating macrophage homeostasis in skeletal muscle upon injury raises the possibility that modulation of miR-155 expression levels or its downstream targets could bring therapeutic benefits in various muscle-wasting diseases. Such treatment might also serve as a means of intervention in treating degenerative muscle diseases. In addition, owing to its molecular nature—small, abundant, and stable—the ability to quickly modulate expression levels using modified small RNA molecules or inhibitors makes miRNA-based intervention an attractive option for treating complex muscle-wasting diseases.

## Materials and Methods

### Mice and genotyping

Bic/miR-155^−/−^ mice in a 129-C57BL/6 mixed background were described previously.^38^ miR-155-KO mice were generated by mating miR-155^+/−^ male and female mice. C57BL/10ScSn-Dmd_mdx/_J mice were purchased from Jackson Laboratory (Bar Harbor, ME, USA). All animals were housed in sterile, pathogen-free isolation cages. Previously described primers for genotyping of miR-155 and *mdx* were used.^42, 78^ All experiments with mice were performed according to protocols approved by the Institutional Animal Care and Use Committees of Boston Children's Hospital.

### Cardiotoxin injury

Cardiotoxin from *Naja Mossambica mossambic*a (Sigma-Aldrich, St. Louis, MO, USA) was dissolved in sterile saline to a final concentration of 10 *μ*M. In total, 50 *μ*l of cardiotoxin were injected with a 27 Gauge needle into one TA muscle; the other muscle was injected with saline as control.

### miRNA mimic *in vivo* transfection

Injection of miR-155 mimics into the TA muscles of young adult mice was adapted from previous reports.^29, 61^ In total, 50 *μ*l of microRNA complex was injected into the TA muscle 12 h after cardiotoxin injection and mice were analyzed at the indicated time points.

### Histological analysis of skeletal muscles

Skeletal muscles were dissected out and fixed in 4% paraformaldehyde and processed for Hematoxylin and Eosin, Sirius Red, and Fast Green staining as previously described.^79^ For immunofluorescent staining, skeletal muscle groups were harvested and freshly frozen in liquid nitrogen cooled isopentane (Sigma-Aldrich) and then cryo-embedded in Tissue-Tek OCT medium (Sakura Finetek Inc., Torrance, CA, USA). Muscles were sectioned on a cryostat at 10 *μ*m thickness and placed on permafrost slides (VWR Scientific, Radnor, PA, USA). Images were taken with a Zeiss SteREO Discovery V8 stereomicroscope.

### Immunohistochemistry and immunofluorescence

Frozen muscle sections were fixed in 4% paraformaldehyde and permeabilized in 0.5% Triton X-100 for 10 min. Sections were incubated with mouse IgG-blocking solution from the M.O.M kit (Vector Lab, Burlingame, CA, USA) according to the manufacturer's protocol. Primary and secondary antibodies were as following: Desmin (1 : 200, Santa Cruz, Dallas, TX, USA), dystrophin (1 : 200, Sigma-Aldrich), Laminin (1 : 500, Sigma-Aldrich), MF20 (1 : 10, DSHB), Pax7 (1 : 100, DSHB), eMHC (1 : 200, DSHB), BA-F18 (1 : 2, DSHB), BAD5 (1 : 2, DSHB), and Rabbit anti *β*-galactosidase (1 : 500, Sigma-Aldrich). FITC-conjugated F4/80 and CD11b (eBiosciences, San Diego, CA, USA) were used for staining macrophage markers in cardiotoxin injured TA muscles. All secondary antibodies were obtained from Invitrogen (Carlsbad, CA, USA) and used at 1 : 500 dilutions. Pictures were taken with a Nikon TE2000 epifluorescent microscope with deconvolution (Volocity; Perkin-Elmer, Waltham, MA, USA) or an Olympus FV1000 confocal microscopy (FV1000, Olympus, Center Valley, PA, USA).

### Primary myoblast isolation, culture, and differentiation

Primary myoblasts were isolated from neonatal mice as previously described.^47^ Primary myoblasts were further enriched by pre-plating 30 min for each passage until ~100% cells were positive for desmin. Primary myoblasts were kept in Ham's F-10 nutrient mixture based growth medium containing 20% fetal bovine serum (FBS), 2.5% chicken embryo extract (USbiologicals, Salem, MA, USA), 5 ng/ml bFGF (Promega, Madison, WI, USA), 100 U/ml penicillin, and 100 *μ*g/ml streptomycin. Differentiation medium (Dulbecco's Modified Eagle's Medium; DMEM containing 2% horse serum) was used to induce primary myoblast differentiation.

### Macrophage proliferation, viability, and migration assays

Murine macrophage cell line RAW 264.7 was purchased from American Type Culture Collection. Cells were maintained in DMEM (high and low glucose, respectively) supplemented with 10% heat-inactivated FBS (Life Technologies, Carlsbad, CA, USA), 2 mM l-glutamine, 100 U/ml penicillin, and 100 μg/ml streptomycin in a humidified incubator at 37 °C under 5% CO_2_. Proliferation of RAW 264.7 cells was measured using Click-iT cell proliferation assay kit (Invitrogen) according to manufacturer's instructions. A final concentration of 30 nM microRNA LNA (locked nucleic acid) inhibitor of miR-155 and negative control oligonucleotide (Dharmacon, Lafayette, CO, USA) were transfected into RAW 264.7 cells using Lipofectamine RNAiMAX (Invitrogen) transfection reagent. After 6 h transfection, the cultures were changed to fresh medium. EdU (5-ethynyl-2′-deoxyuridine, Invitrogen) was added, and 30 h later cells were fixed and harvested for immunohistochemistry analyses. Viability of the cells were assayed using Invitrogen Countess automatic cell counter 72 h after miR-155 or control LNA transfection, with trypan blue dye as indicator of live or dead cells.

Migration assays were performed in Transwell plates (Corning Costar, Tewksbury, MA, USA) with a 6.5-mm diameter and an 8-*μ*m pore size for the membrane.

The RAW 264.7 cells were seeded in six-well plates and transfected with control and miR-155 LNA for 48 h before being transferred into Transwell plates. Fresh medium was added to the bottom well with or without LPS immediately before adding 1 × 10^5^ RAW 264.7 cells into the top well. Plates were incubated for an additional 12 h at 37 °C in a humidified 5% CO2 incubator. Cells in the top wells were wiped off with cotton tips, fixed using 3.7% formaldehyde for 5 min, and stained with DAPI for identifying cell nuclei. The number of cells migrating to the lower membrane surface was then counted, and these data were used for further two-tailed student's *t*-test analysis.

### Single myofiber isolation

Single myofibers were isolated from the EDL muscle as previously described.^80^ For immediate quantification of satellite cells, single fibers were fixed in 4% paraformaldehyde for 10 min at room temperature. Fibers were permeabilized with PBST (PBS with 0.5% Triton X-100) for 15 min and blocked with blocking solution (2% BSA/5% goat serum/0.1% Triton X-100 in PBS) for 1 h at room temperature.

### FACS and MACS analysis

At indicated time points after cardiotoxin injury, TA muscles were dissected, minced, and digested with STEMxyme2 (Worthington Biochemical Corp., Lakewood, NJ, USA). Cell suspension was then serially filtered through 70 and 40 μm nylon meshes (BD Falcon, Franklin Lakes, NJ, USA). All FACS analyses were performed at the Dana-Farber Cancer Institute flow cytometry facilities with a BD FACSAria II SORP UV sorter. Flowjo software was used to analyze the FACS data. Antibodies used include Pacific Blue conjugated anti-mouse CD45 (Biolegend, 30F-11), Alexa 647 conjugated anti-mouse CD68 (Biolegend, San Diego, CA, USA, FA-11), and Brilliant Violet 605 anti-mouse CD206 Antibody (Biolegend, C068C2). For MACS, cells were incubated with FITC-labeled F4/80 and CD11b antibody (eBiosciences) for 30 min in 4 °C and magnetically separated using an EasySep Mouse FITC Positive Selection Kit (Stemcell Technology, Vancouver, BC, Canada). Cell numbers were determined using Countess automated cell counter (Invitrogen).

### RT-PCR, real-time PCR, and taqman assays

miRNAs and total RNAs were extracted using Trizol and were cleaned using miRNeasy kit (Qiagen, Valencia, CA, USA). miRNAs were measured using Taqman MicroRNA Reverse Transcription Kit and Taqman Universal Master Mix Kit (Applied Biosystems, Carlsbad, CA, USA). All SYBR-based real-time PCRs were run on a CFX96 or CFX384 Real-Time PCR machine (Bio-Rad, Hercules, CA, USA) with iScript reverse transcription kit and iTaq supermix (Bio-Rad). A list of SYBR-based real-time PCR primers can be found in the Supplementary Table.

### Western blot analysis

Total muscle protein was extracted using RIPA buffer containing protease inhibitor cocktail (Roche, Indianapolis, IN, USA) and 1 mM PMSF. Protein concentrations were measured by a DC protein assay (Bio-Rad). Western blotting was performed by standard protocol. The following antibodies were used: SOCS1 (1 : 1000, Cell signaling, Danvers, MA, USA), phospho-STAT3 (1 : 1000, Cell signaling), anti-mouse STAT3 (1 : 1000, Cell signaling), *γ*-tubulin (1 : 5000, Sigma-Aldrich), GAPDH (1 : 5000, Sigma-Aldrich). Primary antibody was visualized with either IRDye 680RD goat anti-mouse or IRDye 800CW goat anti-rabbit (LI-COR, Lincoln, NE, USA) on the Odyssey imaging system (LI-COR Biosciences).

### Plasmids, transfection and luciferase assays

The 3'-UTR fragment of mouse SOCS1 containing miR-155 binding sites was cloned into the pMIR-glow vector (Promega, Madison, WI, USA). miR-155 sensor and mutagenesis of the miR-155 binding sites were performed as previously described.^42^ Hek293T cells (CRL-11268;ATCC) were grown in DMEM containing 10% FBS. Transfection was performed with Lipfectamine 2000 reagents (Invitrogen) according to the manufacturer's instructions. For luciferase assays, Firefly and Renila luciferase activity were measured using Dual-Glo Luciferase Assay kit (Promega) according to the manufacturer's instructions. All experiments were performed in triplicate and were repeated at least twice.

### Statistics

Unless otherwise stated, all statistical analyses were performed using unpaired two-tailed student's *t*-test. Data are presented as mean value or percentage change±S.E.M. *P*<0.05 was considered to be statistical significance.

## Abbreviations

BIC: B-cell integration cluster
MEF2A: myocyte enhancer factor 2A
H&E: hematoxylin and eosin
TA: tibias anterior
Quad: quadriceps
GAS: gastrocnemius
pax7, EdU: 5-ethynyl-2′-deoxyuridine paired box protein 7
eMHC: embryonic isoform of myosin heavy chain
TNF-a: tumor necrosis factor alpha
IL-6: interleukin 6
IL-10: interleukin 10
INF-g: interferon gamma
MCP-1/CCL2: monocyte chemoattractant protein-1
RIP3: receptor-interacting protein kinase 3
FACS: fluorescence-activated cell sorting
iNOS: inducible nitric oxide synthase
SCOS1: suppressor of cytokine signaling 1
Jarid2: Jumonji, AT-rich interactive domain 2
SHIP1: phosphatidylinositol-3,4,5-trisphosphate 5-phosphatase 1
TAB2: TGF-beta activated kinase 1/MAP3K7-binding protein 2
LNA: locked nucleic acid
DMD: duchenne muscular dystrophy
CTX: cardiotoxin
