# Supplementary material for: MicroRNA-155 facilitates skeletal muscle regeneration by balancing pro- and anti-inflammatory macrophages
Source: Cell Death Dis. 2016 Jun 9;7(6):e2261–. doi: 10.1038/cddis.2016.165 (PMC5143393; doi:10.1038/cddis.2016.165)
Supplement: Supplementary Information [file cddis2016165x1.pdf]

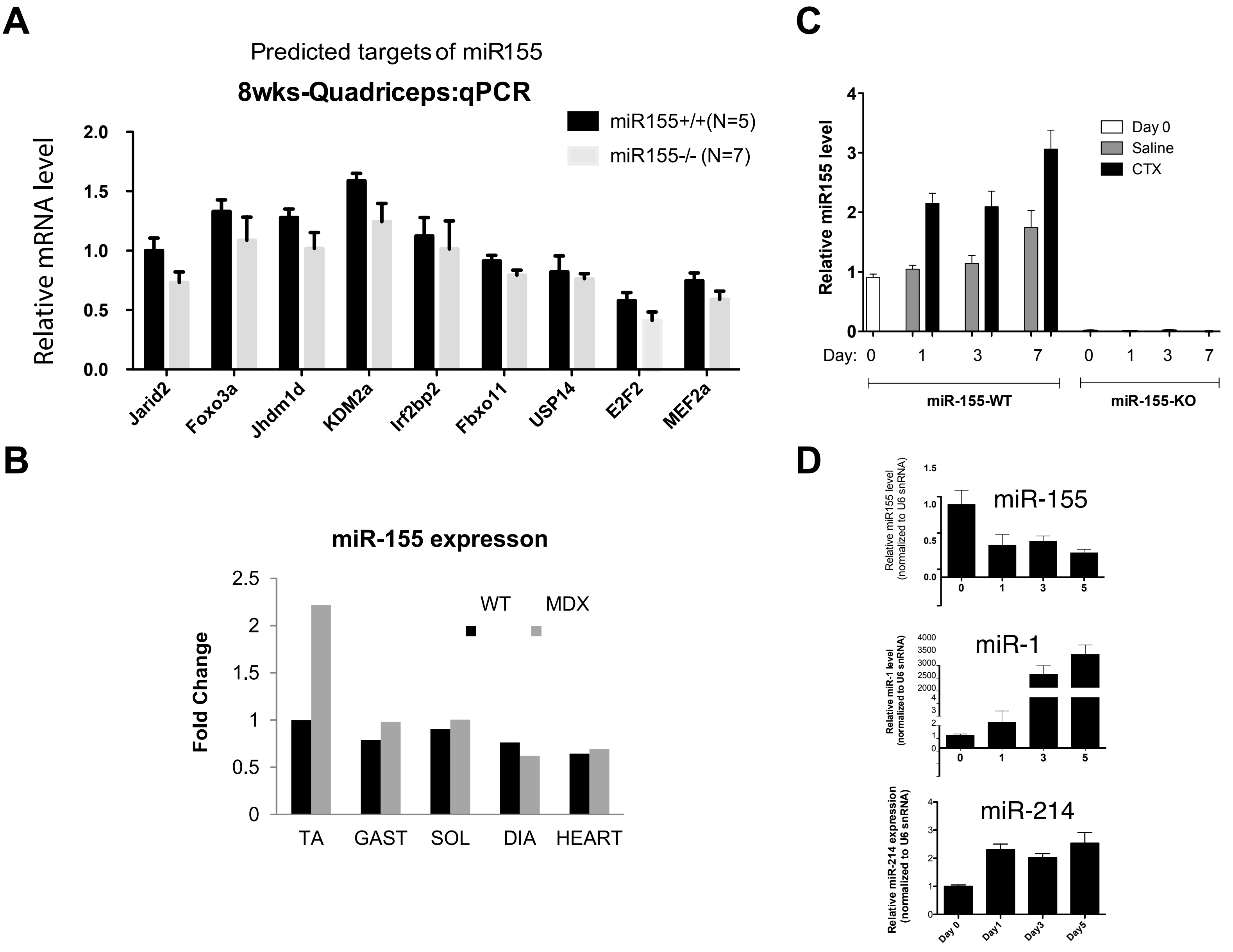

Figure S1: miR-155 and its predicted target mRNAs in C2C12 cells, CTX injected TA muscles, and mdx mice. (A)mRNA levels of previously reported and predicted miR-155 targets in quadriceps muscle from miR-155-WT and miR-155-KO mice, as measured by real-time PCR. Data are presented as Mean ± SEM. (B) Mature miR-155 levels in muscle wild type and mdx mice, measured by Taqman real-time PCR assays. TA, Tibialis Anterior; GAS, Gastrocnenius; Sol, Solues; DIA, Diaphram.
(C) miR-155 expression levels in TA muscle 1, 3, and 7 days after CTX injection, as determined by Taqman real-time PCR assays. N=6 for each group. Data are presented as Mean ±vSEM.
(D) Expression of miR-155 and muscle-specific microRNAs miR-1 and miR-214 during C2C12 differentiation, measured by Taqman real-time PCR. Data are from three independent biological replicates and are presented as Mean ± SEM.

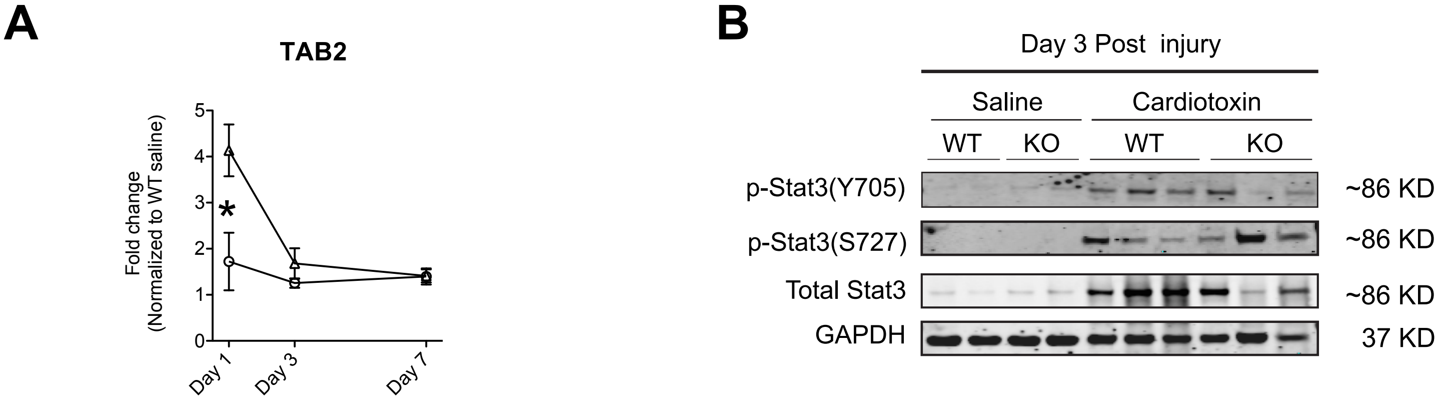

Figure S2: Gene expression and Stat signaling in CTX injected miR-155-KO mice.
(A) TAB2 expression in miR-155-WT and miR-155-KO TA muscle 1, 3 and 7 days after CTX injury, measured by real-time PCR. 4-6 animals of each genotype at each time point were quantified. Data are presented as Mean ± SEM. *p<0.05. (B) Western blot analysis of phospho-STAT3(Y705) and phospho-STAT3(S727) levels in TA muscle of miR-155-WT and miR-155-KO mice 3 days after CTX injection, showing delayed activation of phospho-STAT3(S727) signaling in miR-155-KO mice compared with wild type after CTX injury.

SUPPLEMENTAL TABLE

Genes (syber Green Primers)	Species	Forward Sequence	Reverse Sequence
MyoD	mmu	CGCCACTCCGGGACATAG	GAAGTCGTCTGCTGTCTCAAAGG
myogenin	mmu	AGCGCAGGCTCAAGAAAGTGAATG	CTGTAGGCGCTCAATGTACTGGAT
eMyHC	mmu	TCCAAACCGTCTCTGCACTGTT	AGCGTACAAAGTGTGGGTGTGT
Myf5	mmu	CAGCCCCACCTCCAACTG	GGGACCAGACAGGGCTGTTA
Pax7	mmu	TCTCCAAGATTCTGTGCCGAT	CGGGGTTCTCTCTCTTATACTCC
Myf6	mmu	ATCAGCTACATTGAGCGTCTACA	CCTGGAATGATCCGAAACACTTG
Socs1	mmu	GTGGTTGTGGAGGGTGAGAT	CCTGAGAGGTGGGATGAGG
TAB2	mmu	TTGTTGTATCCAGGTGCATGTT	CCATAAAGATACCTTGTACTCTCCTGA
cebpb	mmu	CAAGTTCCGCAGGGTGCT	CCAAGAAGACGGTGGACAA
MyH1	mmu	AGTCCCAGGTCAACAAGCTG	CACATTTTGCTCATCTCTTTG
MyH2	mmu	AGTCCCAGGTCAACAAGCTG	GCATGACCAAAGGTTTCACA
MyH4	mmu	AGTCCCAGGTCAACAAGCTG	TTTCTCCTGTCACCTCTCAACA
MyH7	mmu	AGTCCCAGGTCAACAAGCTG	TTCCACCTAAAGGGCTGTTG
MCK	mmu	GCAAGCACCCCAAGTTTGA	ACCTGTGCCGCGCTTCT
TshZ3	mmu	ATCAGTGAGACGAGTGACCG	CAGGCTATCAGACACGATGGT
Jarid2	mmu	GGCGAATCTGGTTTTGGGGG	TGGTCCTAGTGCCTTAGCCT
Foxo3a	mmu	ACCGGATACACAGTGGCAAG	CAGCCATCGCGTTGTCAAAT
Jhdm1d	mmu	CGATGTCTGCAAGGACTGGT	CATCGGCACTTGGGAAGACT
KDM2a	mmu	GGTCTGCAGTATCTGCCCTC	CAGCCGGAAGTCAGTCATGT
Irf2bp2	mmu	GCCAGTCGTGCTATCTGTGT	GTAGCGCTCCATAGCCTGAG
Fbxo11	mmu	ACCGGATACACAGTGGCAAG	CAGCCATCGCGTTGTCAAAT
USP14	mmu	TGCTTCGTATTCCTCGGCTG	TCCACATGGCAATTCCATATTTTT
E2F2	mmu	CCCACCACAGACAAGATTGGA	GGTATCGGCTGGGGTATCA
TCF4	mmu	GTAACTGGCGATCTGAGGGG	CACTGCTTACAGGAGGCGAA
Mef2a	mmu	GTGTACTCAGCAATGCCGAC	AACCCTGAGATAACTGCCCTC
IL-6	mmu	CAAAGCCAGAGTCCTTCAGAG	GCCACTCCTTCTGTGACTCC
CD11b	mmu	CAGCAGTGATGAGAGCCAAG	GGCAGGAGTCGTATGTGAGG
MCP-1	mmu	GTTGGCTCAGCCAGATGCA	AGCCTACTCATTGGGATCATCTTG
RIP3	mmu	ATTCATGGAGAATGGCTCC	GAAACGTGGACAGGCCAAA
CD68	mmu	CCAGCTGTTCACCTTGACCT	CAATGATGAGAGGCAGCAAG
INFγ	mmu	GCGTCATTGAATCACACCTG	CTGGACCTGTGGGTTGTTG
TNFα	mmu	CCACCACGCTCTTCTGTCTA	AGGGTCTGGGCCATAGAACT
IL-10	mmu	ATCGATTTCTTCCCTGTGAA	TGGCCTTGTAGACACCTTGG
CD206	mmu	CCACAGCATTGAGGAGTTTG	ACAGCTCATCATTTGGCTCA
INOS	mmu	GGAGCCTTTAGACCTCAACAGA	AAGGTGAGCTGAACGAGGAG
18S rRNA	mmu	TCCGACCATAAACGATGCCG	CAATCTGTCAATCCTGTCCGTGTC

Genes (Taqman Primers)	Species	miRBase ID	Assay Number
miR-155	mmu	mmu-miR-155-5p	002571
miR-1a	mmu	mmu-miR-1a-3p	002222
